# Inhibitory-κB Kinase (IKK) α and Nuclear Factor-κB (NFκB)-Inducing Kinase (NIK) as Anti-Cancer Drug Targets

**DOI:** 10.3390/cells7100176

**Published:** 2018-10-20

**Authors:** Andrew Paul, Joanne Edwards, Christopher Pepper, Simon Mackay

**Affiliations:** 1 Strathclyde Institute of Pharmacy and Biomedical Sciences, 161 Cathedral Street, University of Strathclyde, Glasgow G4 0NR, UK; simon.mackay@strath.ac.uk; 2 Institute of Cancer Sciences, University of Glasgow, Garscube Estate, Switchback Road, Bearsden, Glasgow G61 1QH, UK; Joanne.Edwards@glasgow.ac.uk; 3 Brighton and Sussex Medical School, University of Sussex, Brighton BN1 9PX, U.K.; C.Pepper@bsms.ac.uk

**Keywords:** inhibitory-κB kinase (IKK) α, Nuclear Factor-κB (NF-κB), NF-κB-inducing kinase (NIK), cancer, inflammation, small molecule kinase inhibitor

## Abstract

The cellular kinases inhibitory-κB kinase (IKK) α and Nuclear Factor-κB (NF-κB)-inducing kinase (NIK) are well recognised as key central regulators and drivers of the non-canonical NF-κB cascade and as such dictate the initiation and development of defined transcriptional responses associated with the liberation of p52-RelB and p52-p52 NF-κB dimer complexes. Whilst these kinases and downstream NF-κB complexes transduce pro-inflammatory and growth stimulating signals that contribute to major cellular processes, they also play a key role in the pathogenesis of a number of inflammatory-based conditions and diverse cancer types, which for the latter may be a result of background mutational status. IKKα and NIK, therefore, represent attractive targets for pharmacological intervention. Here, specifically in the cancer setting, we reflect on the potential pathophysiological role(s) of each of these kinases, their associated downstream signalling outcomes and the stimulatory and mutational mechanisms leading to their increased activation. We also consider the downstream coordination of transcriptional events and phenotypic outcomes illustrative of key cancer ‘Hallmarks’ that are now increasingly perceived to be due to the coordinated recruitment of both NF-κB-dependent as well as NF-κB–independent signalling. Furthermore, as these kinases regulate the transition from hormone-dependent to hormone-independent growth in defined tumour subsets, potential tumour reactivation and major cytokine and chemokine species that may have significant bearing upon tumour-stromal communication and tumour microenvironment it reiterates their potential to be drug targets. Therefore, with the emergence of small molecule kinase inhibitors targeting each of these kinases, we consider medicinal chemistry efforts to date and those evolving that may contribute to the development of viable pharmacological intervention strategies to target a variety of tumour types.

## 1. Introduction and Background

### 1.1. Nuclear Factor Kappa-B (NF-κB) Proteins

**Figure 1:**
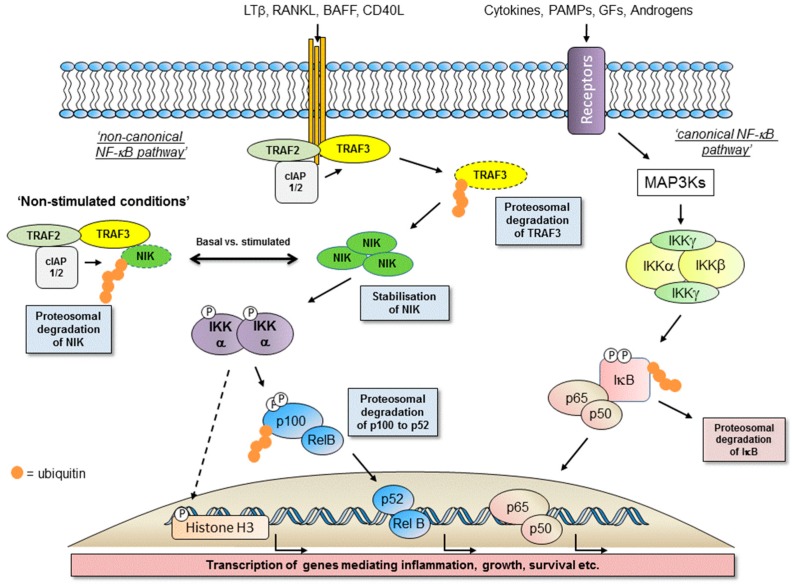
Schematic representation of Nuclear Factor-κB (NF-κB)-inducing kinase (NIK)-inhibitory-κB kinase (IKK) α and IKKβ-mediated cell signalling encompassing the non-canonical NF-κB cascade, canonical NF-κB cascade and potential IKKα-dependent, NF-κB-independent pathways (e.g., by the dashed line).

Nuclear Factor kappa-B (NF-κB), from the Nuclear factor kappa-light-chain-enhancer of B-cells, represents a family of five transcription factors involved in diverse biological responses that underpin phenotypic outcomes of inflammation, modulation of immune responses, cell growth, proliferation, apoptosis and aspects of differentiation and development [1,2,3,4,5]. NF-κB signalling is now appreciated as either canonical (classical) or non-canonical (alternative) pathways via the mobilisation of both homo- and hetero-dimer complexes of these family members (see Figure 1; References [1,2,3,4,5]). Collectively, the NF-κB proteins are five distinct isoforms; RelA (p65), RelB, c-Rel, NF-κB1 (p105/p50) and NF-κB2 (p100/p52) [1,2,3,4,5]. In an inactive state these proteins are typically associated with inhibitory-κB (IκB) proteins, including isoforms of IκBα, IκBβ, and IκBε and in the case of p105 and p100 proteins it is their intrinsic protein structure that maintains them in a self-bound inhibited form by virtue of their C-terminal IκB-like structures (IκBδ and IκBγ respectively) composed of ankyrin repeats [1,2,3,4,5]. Activation and liberation of NF-κB proteins occur typically in response to a number of extracellular ligands, as well as agents that generate a DNA Damage response (DDR), resulting in the nuclear localisation of DNA-binding protein dimers following dissociation from IκB molecules [1,2,3,4,5]. 

### 1.2. Activation of the Non-Canonical NF-κB Pathway

The canonical pathway (reviewed elsewhere in this issue and previously in References [6,7]), can be activated in response to cytokines such as TNFα and IL-1β and pathogen-associated molecular profiles (PAMPs) such as the bacterial endotoxin lipopolysaccharide (LPS) [6,7]. This response is typically rapid and transient, mediated by the classical inhibitory-κB kinase (IKK) complex (IKKα/β/γ) with a requirement for IKKβ-mediated phosphorylation of selected IκB proteins [6,7]. In contrast, activation of the non-canonical NF-κB pathway is relatively slower and over a period of hours results in an IKKα-mediated liberation of predominately p52-RelB dimers to drive gene transcription [1,2,3,4,5,6,7]. This slower response reflects reliance upon protein expression/stabilisation within the upstream components of the pathway (see below). Whilst TNFα and IL-1β have the ability to activate the non-canonical NF-κB pathway, it is typically alternative members of the greater TNF superfamily that drive activation (see References [3,4]). This includes lymphotoxin-β (LT-β), the related tumour necrosis factor superfamily member 14 (TNFSF14) known as LIGHT, TNF-like weak inducer of apoptosis (TWEAK), CD40 ligand (CD40L), Receptor-activator of NF-κB ligand (RANKL) and B-cell activating factor (BAFF), all reviewed extensively elsewhere [1,3,4].

### 1.3. Major Components of The Receptor Activated Non-Canonical NF-κB Pathway

**Figure 2:**
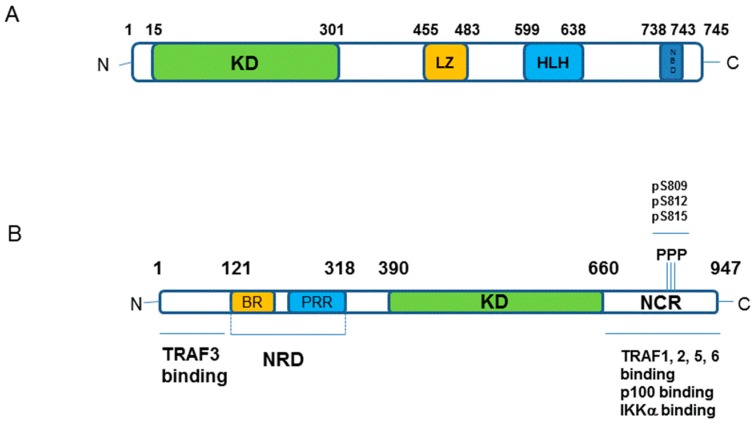
Schematic representation of IKKα and NIK kinase components of the non-canonical NF-κB cascade. (**A**) IKKα protein structure; Key: KD–kinase domain; LZ–Leucine Zipper; HLH–helix-loop-helix; NBD–NEMO Binding Domain. The amino acid numbering of domains and phosphorylation sites (P/pS) as indicated. (**B**) NIK protein structure; Key: NRD–negative regulatory domain containing BR–basic region, PRR–proline-rich region; KD–kinase domain; NCR–non-catalytic region. The amino acid numbering of domains as indicated.

Through a combination of molecular and genetic studies, we now appreciate receptor-mediated-non-canonical NF-κB activation to be built around the paradigm of a TNF super family ligand activating its cognate receptor via recruitment of a sequence of identifiable adaptor molecules of the TNF-Receptor associated factor (TRAF) family, notably TRAF2 and TRAF3, modulators of ubiquitination and associated protein degradation in the form of the cellular inhibitors of apoptosis (cIAPs). These proteins enable engagement and activation of the cellular kinases NF-κB-inducing kinase (NIK; see Figure 2A), the 14th member of the MAP kinase kinase kinase (MAP3K) family, and IKKα (see Figure 2B) to determine the liberation of p52-RelB protein complexes (see Figure 1; References [1,2,3,4,5]). 

The indication and identification of NIK as the first major regulatory component of the non-canonical NF-κB pathway came from studies upon alymphoplasia (aly/aly) mice that display an amino acid substitution (G886R) in the NIK protein structure [8,9,10,11]. These mice showed phenotypic similarities with those with deficiencies or mutations in NF-κB2/p100 (see Reference [1]). Aly/aly mice were characterised as displaying low levels of B lymphocytes, the systemic absence of lymph nodes and Peyer’s patches and disorganised splenic and thymic structure with immune-deficiencies around defective antibody responses [11]. The aly/aly mutation impairs NIK interactions with IKKα via its C-terminal interaction domain (see Figure 2; Reference [11]) and overlaps with the phenotype displayed by NIK-deficient mice [12]. Interestingly, the phenotypes of aly/aly mice are more severe than those of LTβR^−/−^ mice [13], however, upon examination of other TNFSFR ‘knock-out’ models, the involvement of NIK in signal transduction is mediated by other TNFSF receptors [11], thus functioning as a key integrator of ligands activating these receptors. As an outlier, animals that are deficient in IKKα present with a different phenotype; IKKα deficiency is lethal and in developing embryos impacts upon limb, skeletal and skin development [14,15]. Limited development of Peyer’s patches may be a common feature with deficient/mutated NIK and NF-κB2 backgrounds but the striking differences may simply reflect the fact that IKKα functions not only in the non-canonical NF-κB pathway but also in regulating canonical NF-κB signalling [6,7]. That aside, the similarities in phenotype(s) of the aly/aly, NIK-deficient and NF-κB2 (p100/p52)-deficient mice can be extended to include RelB as well as other members of the TNF superfamily of receptor (all reviewed in References [3,4]) with varying degrees of overlap and also identifies the key role on non-canonical NF-κB signalling in mediating immune development, immune cell function, antibody responses and the overall architecture of the lymphatic system. At this broader phenotypic level, it remains to be established whether targeting of aberrant NIK-IKKα signalling pharmacologically will generate, in-part or wholly, similar outcomes in vivo that will have potential toxicity implications for any therapeutic designed to dampen aberrant NIK-IKKα and/or non-canonical NF-κB signalling. 

In a cellular setting, under resting non-stimulated conditions, NIK is maintained at a low expression level based upon NIK-focussed proteasomal degradation. However, upon receptor activation NIK is stabilised, protein expression is increased to enable pathway activation [16]. It is TRAF3 that acts as the crucial regulator of NIK expression by controlling the extent of its proteasome-mediated degradation [16]. TRAF3 functions as an adaptor protein to bind NIK and enable its K-48-linked ubiquitination and degradation [16]. It is by virtue of TRAF3-NIK complexes forming a larger molecular weight protein complex with TRAF2 and cIAPs 1/2 that enables ubiquitin ligase enzymatic activity to regulate NIK status by maintaining a low level of expression [17,18,19,20]. Upon receptor activation, the focus of proteasome-mediated protein degradation switches from NIK to that of TRAF2 and TRAF3, which stabilises NIK expression to initiate the sequence of signalling events toward p100 processing [20]. The cIAP proteins that function as ubiquitin ligases to ubiquitinate NIK then target TRAF3 for degradation to increase NIK protein levels. Cell-based studies have identified that cIAPs interactions with NIK are TRAF3- and TRAF2-dependent, although redundancy exists with respect to TRAF2 and TRAF3 mediated stabilisation of NIK [20], likely reflecting defined regions of interaction in the NIK structure (see Figure 2B). TRAF3 binds to NIK independently of TRAF2 whilst TRAF2 itself forms interactions with cIAP1/2. [20]. Interestingly, cIAP 1 and cIAP2 also have redundant functions; single deletions in murine embryonic fibroblast have little impact on non-canonical NF-κB pathway activation [20] whereas double mutants in a murine B lymphocyte background results in a constitutive, elevated activation of this pathway [21].

Upon NIK protein stabilisation, as the first component of the non-canonical NF-κB pathway, it catalyses the phosphorylation of IKKα and supports IKKα recruitment to and phosphorylation of p100 to drive subsequent p100 ubiquitination and proteasome-mediated degradation to liberate p52 [16]. Under basal conditions, p100 exists typically in dimer complexes with RelB and upon stimulated degradation generates p52-RelB dimers able to translocate to the nucleus to initiate the transcription of distinct genes (see Figure 1; see below).

**Figure 3:**
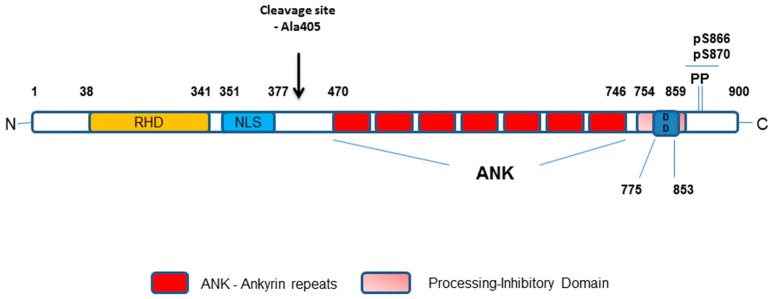
Schematic representation of the p100/p52 protein domain structure. Key: RHD–Rel Homology Domain; NL–Nuclear Localisation Sequence (Glycine-Rich Region); ANK–Ankyrin repeats; DD–Death Domain. Amino acid numbering of domains as indicated.

Both NIK and IKKα play critical roles in the phosphorylation of p100 to liberate mature p52-RelB protein dimers. However, whilst IKKα is now viewed as the key modulator of p100 phosphorylation there is a co-dependence on NIK to deliver coupled phosphorylation and processing of p100 to generate mature p52 that is transcriptionally active [22]. In transfected cells, NIK can stimulate the phosphorylation, ubiquitination and processing of p100 [23,24], however recombinant NIK itself does not display any phosphorylation of p100 in vitro [24,25]. In a cell-based setting, NIK mediates downstream signalling by engaging and activating IKKα resulting in the phosphorylation of the C-terminal region of p100 [25] and this is independent of the other IKK isoforms, β and γ, associated with canonical NF-κB activation [26,27]. Whilst IKKα phosphorylates p100 and regulated non-canonical NF-κB activation alone, it is not as effective at inducing p100 processing as NIK [23]. With these observations, further studies then identified NIK to have a critical role in regulating p100 processing via the recruitment of IKKα to and binding with p100 as a protein substrate [22]. Collectively, NIK-IKKα interaction with p100 results in the phosphorylation of p100 at specific serine residues, which phospho-specific antisera have identified as primarily Ser868/870 as the major phosphorylation sites of p100 in a cell-based setting [24]. These sites are components of the phospho-degron within the p100 C-terminal NIK-responsive domain (NRD) and, when phosphorylated, lead to βTrCP binding as part of the SCF^βTrCP^ ubiquitin ligase complex that drives the eventual processing of p100 [24]. This overcomes the inhibitory influence of the Ankyrin Repeat Domains (ARD) and death domain (see Figure 3) within the p100 structure to generate p52. These specific serines are phosphorylated in cells treated with non-canonical NF-κB activators and inhibition of receptor-mediated phosphorylation of these sites (Ser866/870), detected using phospho-specific antisera, has been reported with first generation IKKα-selective inhibitors in a cell-based setting [28]. Going forward, these inhibitors may help gain a better understanding of the agonist-driven changes in protein-protein interactions and phosphorylation events associated with NIK-IKKα engagement with p100 and thereafter recruitment of components of the SCF^βTrCP^ complex that enables successful processing to mature p52 by cleavage at amino acid Ala405 (see Figure 3).

### 1.4. IKKα-Dependent, NF-κB-Independent Signalling Integrates with IKKα-Dependent Non-Canonical NF-κB Signalling to Contribute to Global Cellular Transcription

Independent of the non-canonical NF-κB pathway, a number of studies in diverse settings have identified that at the NIK-IKKα kinase level there are also examples of signal bifurcation. These can be dependent on differing extracellular conditions (e.g., Reference [29]) and demonstrate that p100 is not the only substrate for IKKα-mediated phosphorylation. IKKα, via catalysed phosphorylation, regulates directly a number of cellular proteins that then either directly or indirectly regulate cellular transcription (previously considered in References [6,7]). This includes transcription factors distinct from the NF-κB family, for example E2F1 [30,31], β-catenin [32], CBP [33], as well as the suppressors of transcription such as the silencing mediator for retinoic acid and thyroid hormone receptor (SMRT) [34] and cell cycle regulator cyclin D1 [35]. Additional substrates also include the protein inhibitor of activated STAT1 (PIAS1) as a modulator of transcription/inflammation [36], the oestrogen (ER) [37] and androgen receptors (AR) [38] of the steroid hormone family receptor along with their associated steroid receptor co-factor (SRC)-3 [37,39,40] and Aurora kinase A [41,42] that contributes to the mitotic process. Direct modulation of the status of these proteins by IKKα has bearing on the transcription of additional regulatory proteins such as p53 [43,44] and EZH2 [44] and additional mitotic kinase Polo-like kinase (PLK) 4 [45]. IKKα, therefore, serves as a key switch in the coordinated regulation of both NF-κB-dependent and NF-κB-independent gene transcription and this underpins the outcomes associated with events that initiate and/or perpetuate the development of acquired characteristics, or phenotypes, we now recognise as cancer ‘hallmarks’ as identified and defined by Hanahan and Weinberg [46,47]. The transcriptional modulation driven by IKKα-mediated signalling, divulged using a number of experimental approaches such as genetic deletion and reconstitution [48,49], siRNA ‘run-down’ [35] and over-expression strategies [50], may be in excess of 200 genes and these gene/protein induction/repression events support the acquisition of characteristics of specific ‘hallmarks’, particularly the ability of tumours to ‘sustain proliferative signalling’, ‘resist cell death’, ‘evade growth suppressors’ and encourage ‘genomic instability and mutation’. Perhaps more striking is the role of IKKα in regulating genes/protein that helps to underpin the phenotypes associated with longer term tumour development: ‘inducing angiogenesis’ and ‘activating invasion and metastasis’ by way of regulating cytokine (e.g., IL-1β, IL-6 [48,49]) and chemokine (e.g., CCL19, CCL21, CXCL12, CXCL13 and BAFF [27,51,52]) induction and modulation of adhesion molecule (e.g., VCAM; References [48,49,50]), maspin [50,53] and MMPs [50] expression in different cellular/tissue situations. It is also evident that in particular sub-types of cancer the acquisition of a specific mutation, C250T in the hTERT promoter [54], that supports tumour reactivation has identified the potential for tumours to become ‘addicted’ to IKKα-mediated non-canonical NF-κB signalling thus ‘enabling replicative potential’. Collectively, perturbation of this enzyme, and perhaps NIK too, could have wide-ranging effects on the multiple hallmarks of tumour cells described above. Moreover, given the impact of IKKα in regulating major cytokine, chemokine and matrix metalloproteinase isoforms, intervention against this enzyme may have significant effects on tumour-stromal communication and matrix composition within the tumour microenvironment and define a better understanding of ‘tumour-promoting inflammation’. In this context, the emergence of selective small molecular kinase inhibitors will allow any potential regulatory contribution(s) made by IKKα and/or NIK to be explored both in an in vitro setting, with isolated homogeneous cell populations and in more complex co-culture and in vivo settings.

### 1.5. Signalling Complexities

Additional complexities to the regulation of IKKα-dependent, NF-κB-dependent and -independent gene transcription are also now apparent in the cancer setting, as we now appreciate that this transcriptional process is not wholly driven by receptor-mediated activation. For both solid tumour (e.g., pancreatic adenocarcinomas) and haematological settings (e.g., multiple myeloma) constitutive activation of IKKα-mediated signalling has been reported as a result of modulation of expression of upstream TRAF and cIAP components in the pathways or mutation in these very same components that ultimately results in constitutive activation of the pathway in the absence of agonist (see sections below). Furthermore, a truncated p45 form of IKKα has been identified in a number of colorectal cancers [55,56], particularly those with a recognised B-Raf^V600E^ mutant background. This drives p45 IKKα-mediated nuclear signalling in a TNF superfamily member-independent manner (see below) and so brings additional mechanistic and transcriptional diversity to tumour development, which has implications for potential intervention therapeutically. 

Given the roles that IKKα and NIK have in the transcriptional induction and responses that underpin the development and maintenance of key phenotypic outcomes we associate with ‘hallmarks’ of cancer described above, we consider the status of these proteins in both solid tumours and haematological cancers and how they may represent potential drug targets in each. Furthermore, we review the developed and emerging medical chemistry focused on targeting their enzymatic and cellular activities as potential routes to clinical intervention. 

## 2. IKKα in Solid Tumours

In recent years, the role of the non-canonical NF-κB pathway and IKKα within it have increasingly been implicated in the development and progression of multiple solid tumours. The non-canonical NF-κB pathway has been associated with poor prognosis in glioblastoma [57] and mouse orthotopic models have demonstrated that up-regulation of this pathway is associated with an aggressive glioblastoma subtype [58]. In prostate cancer, nuclear localisation of RelB is associated with higher grade tumours [59] and the treatment of prostate cancer cells with androgens induces accumulation of nuclear p52 [60]. In addition, silencing of IKKα reduces androgen receptor activity and gene expression, providing evidence that IKKα is associated with prostate cancer growth [60]. Therefore, IKKα may be an attractive target for prostate cancer as the androgen receptor is the main driver of prostate cancer proliferation and inhibition of cell death.

In pancreatic cancer, the non-canonical NF-κB pathway is constitutively activated and associated with increased cell proliferation [51,61]. NIK is elevated in pancreatic cancer and associated with increased proliferation [61,62] and up-regulation of RelB and p52 are associated with mutated KRAS pancreatic cancer [63] with IKKα dependent gene expression being observed [51]. In gastrointestinal tumours NF-κB2^DCT/DCT^ mice develop tumours spontaneously, providing evidence that p100/p52 drives oncogenesis in this setting [64]. In renal cancer, members of the non-canonical NF-κB pathway are associated with poor prognosis, increased disease stage and decreased local inflammation [65] and NIK is associated with increased cell growth and tumourigenicity in ovarian cancer [66]. In lung cancer, NIK is elevated [3] and RelB associated with shorter overall survival, differentiation, tumour invasion, lymph node metastasis, distant metastasis and ‘tumour, node, metastasis’ (TNM) stage [67]. In bladder cancer, up-regulation of RelB and p52 correlate with histological grade, stage and lymph node metastasis [68]. 

There are also numerous studies investigating NIK and IKKα in breast cancer. NIK is reported to be associated with activation of NF-κB in basal-like breast cancer [27] and IKKα, RelB and p52 are associated with decreased cancer-specific survival in ER-positive breast disease [69,70]. Bcl3 can form a DNA-binding complex with p52 and has been observed as over expressed in breast cancer samples [27]. IKKα is demonstrated to play an essential role in the proliferation of mammary epithelium and it is therefore not surprising that aberrant IKKα signalling has been reported in breast cancer [71]. Yang reports that in HER2 positive epithelial cells nuclear IKKα can promote progression to tumourigenesis via p27 [72]. In transgenic mice, overexpression of p100/52 result in a delay of mammary gland development, which is accompanied with over expression of cyclin D1, MMP2, MMP9 and COX-2 expression and results in the mice developing multiple tumours [73]. In addition, constitutive RANK signalling causes elevation of non-canonical NF-κB signalling in breast cancer cell lines, which subsequently stimulates cell proliferation via increased transcription of cyclin D1 [74,75,76] and nuclear IKKα expression is observed in invasive ductal carcinoma and associated with disease-free survival [20]. Immuno-histochemical studies have demonstrated that the p52 subunit is expressed at a higher level in the breast cancer tissue compared to normal adjacent tissue [77] and Western blots of nuclear fractions extracted from cancerous and adjacent normal breast tissue confirm an increase in p52 levels in the tumour cells [77]. This is accompanied by an increase in mRNA levels of p52, Bcl-3 and cyclin D1, all genes regulated by IKKα [77]. In addition, IKKα has been demonstrated in cervical, lung, prostate and pancreatic cell lines to regulate mTORC1 and mTORC2 which control tumour cell proliferation [78]. Taken as a whole, there is now a large body of evidence to support the role of the IKKα-NF-κB non-canonical pathway in the development and progression of solid tumours.

### IKKα Signalling Independent of NF-κB Pathways in Solid Tumours

In addition to the role IKKα plays in NF-κB pathways, it is also reported to have a role independent of both the canonical and non-canonical NF-κB pathways. One such observation is that IKKα accumulates in the nucleus, where it can phosphorylate a variety of substrates including histone H3, SMRT and nuclear co-repressor (NCoR) [79]. In colorectal cancer, IKKα phosphorylates SMRT, resulting in increased expression of Notch-dependent genes [79]. In addition, IKKα has been reported to be associated with NOTCH activation in the presence of anti-oestrogens in breast cancer, resulting in up-regulation of ER-dependent gene expression and providing a mechanism for hormone resistance in an NF-κB independent manner [80,81]. Bennett reported that IKKα expression and not NIK or RelB is associated with recurrence in Luminal A breast cancer, suggesting it is independent of the non-canonical NF-κB pathway [70]. In a second cohort of patients who received tamoxifen, the authors reported that cytoplasmic IKKα was associated with disease-free survival and recurrence-free survival on tamoxifen in Luminal A disease, which may predict patients likely to develop resistance to tamoxifen or IKKα targeted therapies [70] again supporting a role for IKKα in tamoxifen-resistant breast cancer. In contrast however, Roseweir reported in the Tamoxifen and Exemestane Adjuvant Multinational (TEAM) clinical trial cohort that low IKKα expression is associated with increased risk of recurrence on sequential tamoxifen/exemestane therapy, suggesting that the role of IKKα in hormone therapy resistance may change depending on the mechanism of action of the therapy the patient receives [82]. 

In gastric cancer, *Helicobacter pylori*-mediated NF-κB activation is thought to occur via an IKKα-linked pathway that is independent of the non-canonical NF-κB pathway but involves both IKKα and NIK to up-regulate inflammatory infiltrate and promote tumourigenesis [83]. Studies of IKKα independent of the non-canonical NF-κB pathway in colorectal cancer and cutaneous squamous cell carcinoma have centred on a truncated form of IKKα (p45 IKKα) that is constitutively active and specifically resides in the nucleus [55,56]. Bennett observed that nuclear IKKα in breast cancer has a stronger predictive power than cytoplasmic IKKα and proposed that this could be due to detection of the truncated activated form of p45 IKKα as the antibody employed was unable to distinguish between full length IKKα and the truncated p45 IKKα form [70]. Other studies of IKKα signalling independent of the non-canonical NF-κB pathway in colorectal cancer provide additional evidence that IKKα binds to Notch-dependent gene promoters to upregulate them and release chromatin-bound SMRT, which can be restored by inhibition IKKα and results in colorectal cancer xenografts shrinking in size [56]. It has been reported that the truncated p45 IKKα, forms a complex with full length IKKα and NEMO and is responsible for regulating phosphorylation of SMRT and histone H3 in an NF-κB-independent fashion. In addition, p45 IKKα may be phosphorylated in a TAK1-dependent but in an NF-κB-independent manner in BRAF^V600E^ mutant colorectal tumours [56], so supporting a role for nuclear IKKα independent of non-canonical NF-κB signalling. 

The nuclear role of IKKα is consistently reported as being independent of NF-κB, by activating alternative pathways such as NOTCH [84]. This has been observed in breast cancer, skin cancer and osteosarcoma [85]. In liver cancer Hepatitis B virus X protein down-regulates maspin expression via nuclear IKKα resulting in chemoresistance, suggesting that targeting IKKα could re-sensitise HCC tumours to chemotherapy [86]. In transgenic adenocarcinoma of the mouse prostate (TRAMP) models of prostate cancer, IKKα can translocate to the nucleus to promote metastasis and development of castrate-resistant disease in a maspin dependent manner, which is accompanied by a local inflammatory response [87]. Similar to breast cancer, in prostate cancer nuclear IKKα appears to provide a mechanism for hormone resistance as IKKα is associated with the development of castrate-resistant prostate cancer [53] and deletion of BAG3 which is required for IKKα nuclear translocation delays development of castrate-resistant disease [88]. However, it is unknown if nuclear IKKα associated with castrate-resistant prostate cancer is full length or the truncated p45 form. Overall, although in breast and prostate cancer there is currently no evidence of truncated p45 IKKα, there does appear to be an NF-κB independent role for nuclear IKKα in a variety of solid tumours.

## 3. IKKα Association with Hallmarks of Cancer in Human Tumours

As mentioned previously, the NF-κB pathways regulate the transcription of a wide range of genes involved in the inflammation, proliferation and apoptosis. Many of these processes are hallmarks of cancer [46,47] and NF-κB has been hypothesised to be a link between inflammation and tumourigenesis. Whether IKKα functions as a member of the non-canonical NF-κB pathway or in its NF-κB-independent roles, it is clear that it is involved with multiple hallmarks of cancer including key roles in innate and adaptive immune responses, cell survival, cell death and inflammation [89,90]. The non-canonical NF-κB pathway has key roles in regulating processes including production of lymphoid organs (responsible for B and T lymphocyte production), B-cell development and survival, dendritic cell function and bone metabolism [91] and has been reported to promote development and progression of cancers via promotion of inflammatory infiltrate. Studies in mouse models have demonstrated that mice with a dominant-negative, catalytically-inactive IKKα, have reduced adenoma formation, smaller colorectal tumours with a lower proliferation index when treated with a carcinogen and this was associated with increased recruitment of macrophages and other immune cell types [92]. In skin cancer studies, IKKα has been demonstrated to induce inflammation-related genes [93]. In an additional study using a model of peritoneal metastasis in immune-competent mice, intraperitoneal injection with IκBα suppressed colon cells induced an M1-like macrophage phenotype, with reduced liver and peritoneal metastases in vivo. This was associated with increased intra-tumoural activated CD4^+^ and CD8^+^ T cells and reduced angiogenesis [92], demonstrating that NF-κB pathways work with local inflammatory infiltrate to promote colorectal cancer progression. In renal cancer, the inflammatory effects of the NF-κB pathway have mainly been attributed to the canonical p65/p50 subunits in conjunction with STAT3. However, NIK and RelB have previously been shown to be crucial for B-cell development [2], suggesting that the non-canonical NF-κB pathway also plays a role and that RelB can modulate local inflammatory infiltrate in renal cell carcinoma. IKKα is also associated with promoting expression of pro-inflammatory cytokines such as IL-8 in prostate cancer [94]. 

Kong suggests that IKKα can be phosphorylated via deleted in breast cancer 1 (DBC1) to regulate B cell activation via RelB activity and causing increased cell proliferation in mice [95]. However, Bennett demonstrated that silencing of IKKα expression only impacted cell proliferation and apoptosis in ER-positive MCF7 breast cancer cells and not in ER-negative MDA-MB-231 cells [70]. In addition, polymerase chain reaction (PCR) array-based gene transcriptional profiling experiments demonstrated that reducing cellular IKKα expression had a significant impact on the increased expression of genes associated with the induction of apoptosis, in particular, BAK1 and BBC3, providing evidence that IKKα is involved regulating both cell proliferation and apoptosis in ER-positive breast cancer. This is supported by Park who reported that IKKα is involved in regulation of oestrogen-dependent genes such as *cyclin D1* and *c-myc,* which also control cell proliferation [39] and Dan demonstrates that IKKα via mTORC can induce cell proliferation in cervical, lung, prostate and pancreatic cell lines [78] and in basal cell carcinoma IKKα is associated with proliferation and EMT [93]. Studies in vitro also demonstrate that ovarian cancer epithelial cell proliferation, migration and an invasive phenotype of the cancer were promoted via up-regulation of IKKα [20]. In addition, NIK levels have been associated with regulating both cell proliferation and apoptosis in colorectal cancer, demonstrating that the non-canonical NF-κB pathway is involved in cell viability and tumour growth [96].

In conclusion, when this evidence is considered in the context of the hallmarks of cancer, the main function of IKKα is to regulate inflammation, proliferation and apoptosis across a range of solid tumours to promote development and progression of cancer. 

## 4. NF-κB in Haematological Malignancies

Aberrant NF-κB signalling and associated gene transcription that modulate cellular processes involved in the initiation, maintenance and progression of human malignancies are also common to haematological cells and cancers. In this regard, many B-cell leukaemias and lymphomas display abnormal NF-κB activation, implicating this family of transcription factors in these diseases and suggesting these proteins may represent promising therapeutic targets. In addition, it is now appreciated that conventional cytotoxic agents can increase NF-κB activation, contributing to the development of drug resistance via a number of distinct mechanisms. Therefore, inhibitors of global NF-κB signalling, as well as those that target NIK-IKKα-mediated signalling, may prove clinically useful as single agents and also to re-sensitise patients to chemotherapeutic drugs. Understanding of how pharmacological perturbation of canonical NF-κB signalling versus NIK-IKKα-dependent non-canonical NF-κB signalling and/or NF-κB-independent signalling in this setting is in its infancy. Consequently, future comparative analysis with emerging selective small molecule inhibitors will undoubtedly help clarify the relative contribution of these individual pathways to differing sub-types of these forms of cancer. A number of IKKβ inhibitors have been developed [97,98,99] but to date, no selective inhibitors of either IKKβ or IKKα have entered the haematological clinical arena. However, given the frequency of genetic mutations in the non-canonical NF-κB pathway and its critical role in tumour microenvironmental signalling, IKKα, and NIK, represent attractive anti-cancer targets.

In the haematological setting, the non-canonical NF-κB pathway can be activated by a number of different ligands, including BAFF, LTβ, RANKL, CD40L and CD30L [26,27,100,101,102]. The binding of these ligands to their cognate receptors triggers the assembly and activation of the non-canonical NF-κB cascade described earlier [1,2,3,4,5,103]. Again, mature RelB/p52 dimers translocate into the nucleus to initiate the transcription of their target genes. Although it is tempting to consider the two NF-κB pathways as separate, there is cross-talk between them as the canonical NF-κB pathway regulates levels of p100 and RelB [103]. Indeed, activation of both canonical and non-canonical NF-κB pathways have been implicated in haematological malignancies but the underlying causes of the NF-κB dysregulation are diverse even within specific tumour types. Genetic rearrangements, mutations and copy number alterations of NF-κB or IκB members or in genes encoding upstream components of the signalling pathways have all been described in the literature. Beyond the genetic causes, there is now clear evidence that the tumour microenvironment(s) play a critical role in maintaining NF-κB signalling, which is often aberrantly enhanced by the increased secretion of cytokines/chemokines, the persistence of autocrine or paracrine signalling and/or the over-stimulation of immune receptors. 

Chronic lymphocytic leukaemia (CLL) is the commonest leukaemia in Europe and North America. It is characterised by the accumulation of mature-looking CD5^+^/CD19^+^ B lymphocytes in the peripheral blood, bone marrow, and lymphoid tissues [104]. NF-κB is constitutively activated in many CLL patients and this is associated with more aggressive disease [105,106]. A number of recurrent genetic mutations in NF-κB-associated genes have been described in CLL. The most common of these is an inactivating mutation in NFΚBIE that encodes IκBε, a negative NF-κB regulator. These NFΚBIE aberrations are found in approximately 7% of CLL cases and predominantly occur in poor-prognostic subgroups. This may be causal as mutations in NFΚBIE result in increased nuclear translocation of RelA [107]. NOTCH1 mutations occur at an even higher frequency in CLL (~11%). These activating mutations are associated with poor response to chemotherapy [108] and this may be caused by NOTCH1-mediated NF-κB pathway activation [109,110,111]. BIRC3 mutations are found in a smaller proportion of CLL patients (~4%) but they impact upon the non-canonical NF-κB pathway due to the premature truncation of the BIRC3-encoded protein product, cIAP2, resulting in the loss of its E3 ubiquitin ligase activity that is essential for NIK proteasomal degradation. As a consequence, NIK levels increase leading to the phosphorylation of NF-κB2, the processing of p100 to p52 and the constitutive activation of non-canonical NF-κB signalling [112]. Importantly, BIRC3 mutations are associated with a loss of sensitivity to chemotherapy and poor prognosis [113]. Finally, activating mutations in myeloid differentiation primary response gene 88 (MYD88) are found in approximately 3% of CLL cases; these mutations lead to constitutive toll-like receptor (TLR) signalling and an increase in the T-cell attracting chemokines CCL3 and CCL4 [114]. This may be particularly important in the context of the lymphoid niche where T-cell help appears to promote CLL cell proliferation.

In addition to the genetic causes of NF-κB dysregulation in CLL, it is now understood that the lymph node microenvironment plays a critical role in modulating the natural pathology of this disease. Signalling via the B-cell receptor (BCR), toll-like receptors (TLR) and CD40, as well as the engagement of the BAFF and a proliferation-inducing ligand (APRIL) receptors TACI, BAFF-R and BCMA, create a pro-survival, pro-proliferative niche mediated, at least in part, by NF-κB activation [115,116]. The importance of this microenvironment is perhaps best exemplified by the remarkable clinical effects of the Bruton’s tyrosine kinase inhibitor ibrutinib. Treatment with this drug results in a marked tissue redistribution effect with leukaemia cells being excluded from the lymphoid tissues [117]. The partitioning of the tumour away from the sites of increased NF-κB signalling results in durable remissions, an effect that is reversed on drug withdrawal. 

Marginal zone lymphomas (MZL) can be sub-divided into three distinct groups: splenic (sMZL), nodal (nMZL) and mucosa-associated lymphoid tissue (MALT) lymphomas. Both sMZL and MALT tumours commonly display activation of both canonical and non-canonical NF-κB pathways. The t(11;18)(q21;q21) translocation represents the most frequent genetic alteration in MALT lymphomas (~18% of all cases). The translocation generates a cIAP2-MALT1 fusion product which triggers TRAF6-dependent ubiquitination of NEMO and the activation of the canonical NF-κB pathway [118]. In addition, the auto-oligomerisation of cIAP2-MALT1 promotes the recruitment of NIK and its subsequent cleavage by the MALT1 protease domain produces a degradation-resistant NIK kinase and constitutive non-canonical NF-κB signalling [119]. Aberrant NF-κB activation is found in 30–40% of sMZL patients. Again, this can involve dysregulation of the canonical and non-canonical NF-κB pathways. For example, inactivation of the negative regulator *TNFAIP3* (A20) by non-sense or frame-shift mutations causes increased activation of NF-κB signalling. In addition, CARD11 coiled-coil domain mutations promote spontaneous CARD11 multimerisation and IKKβ kinase activation [120]. In terms of non-canonical NF-κB disruption, BIRC3 mutations, similar to those found in CLL, affect NF-κB activation by stabilising NIK. Alternatively, mutations in TRAF3 lead to the stabilisation of NIK [121].

Diffuse large B-cell lymphomas (DLBCL) are the most common types of non-Hodgkin lymphoma. They are divided into three molecular sub-types: ABC (activated B-cell), GCB (germinal centre B-cell) and PMBL (primary mediastinal B-cell lymphoma). Initial evidence for the role of the canonical NF-κB pathway in DLBCL came from gene expression profiling studies, which showed enrichment for NF-κB target genes in the ABC sub-type. This group has the worst prognosis implicating NF-κB as a modulator of clinical outcome in DLBCL [122]. Constitutive NF-κB activation in the ABC sub-type can result from mutations in components of the BCR signalling cascade, which results in chronic BCR activation. These mutations often occur in the immunoreceptor tyrosine-based motif (ITAM) but also in the coiled-coil domain of the CARD11/CARMA1 gene [123]. Finally, MYD88 gene mutations are found in approximately 30% of the ABC sub-type resulting in spontaneous activation of the downstream IRAK complex and NF-κB activation [124]. The non-canonical NF-κB pathway is also aberrantly dysregulated in 10–15% of DLBCL cases due to TRAF2 and TRAF3 mutations [125] and consequently identifies a sub-population of tumours that may be targetable via NIK and/or IKKα.

Multiple myeloma (MM) is an incurable plasma cell malignancy accounting for approximately 13% of all haematological cancers. Disease progression involves clonal expansion of transformed plasma cells in the bone marrow. Overall, genetic abnormalities leading to constitutive NF-κB activity have been found in approximately 20% of MM patients and 40% of MM cell lines [126,127,128]. Most of the genetic abnormalities relating to NF-κB dysregulation in MM involve the non-canonical NF-κB pathway including aberrant expression of NIK, CD40, TRAF2, TRAF3, transmembrane activator and CAML interactor (TACI) and cIAP1/2 [126,127]. In these studies, the majority of MM cases possessed overexpression of the positive NF-κB regulators NIK, TACI and CD40, or reduced or silenced activity of the negative NF-κB regulators TRAF2, TRAF3 and cIAP1/2. All of these phenotypes contribute to increased NF-κB signalling, with a preference towards non-canonical NF-κB signalling [127,128]. Furthermore, targeted shRNA-mediated run-down of NIK abrogates non-canonical NF-κB activity and generates toxicity to NIK-overexpressing MM cells [126], thus suggesting pharmacological intervention against NIK may be a future therapeutic strategy [126]. In addition, other less common genetic abnormalities that also lead to constitutive NF-κB signalling in MM have been identified. These included a high expression of the NFΚB1 gene (p105) and abnormalities within the NFΚB2 gene (p100), which results in increased canonical and non-canonical NF-κB signalling, respectively [126,127,128].

Although genetic abnormalities can explain some of the high NF-κB activity in MM, it is likely that a substantial portion of the NF-κB signalling in this disease arises as a consequence of interactions within the bone marrow microenvironment [128]. One such mechanism for NF-κB activation is via CD40-CD40L interactions [100,129]. CD40 is a cell surface marker not usually expressed on normal plasma cells but has been shown to be increased in the early stages of MM [130]. Furthermore, blocking the interaction of CD40 with CD40L decreases NF-κB activation [126]. This results in the inhibition of IL-6 and vascular endothelial growth factor (VEGF) secretion, which in turn leads to growth arrest and cell death of MM cells [131]. Furthermore, the bone marrow stromal cells (BMSC) found in the MM tumour microenvironment have also been found to express high levels of NF-κB activation that helps to support the proliferation, survival and drug resistance of malignant plasma cells within the bone marrow niche [132]. Adherence of MM cells to BMSCs induces NF-κB-dependent cytokine transcription and secretion of TNFα, IL-6, VEGF, RANKL and BAFF, to promote MM cell survival and growth through MM cell NF-κB activation [133,134].

Although normal plasma cells do not usually express RANKL, MM cells can gain the expression of this ligand [135] and interaction with RANK-expressing osteoclasts mediates their differentiation and activation through activation of NF-κB [136]. Inhibition of NF-κB signalling blocks osteoclastogenesis, which indicates the role of NF-κB in the RANKL-RANK signalling pathway and in establishing the bone-destructive microenvironment that occurs in MM [137]. NF-κB activation in MM can also result from BAFF and APRIL. For example, interference with BAFF signalling significantly reduces plasma cell numbers, which suggests that activation of NF-κB by BAFF contributes to the survival of plasma cells [138]. MM cells highly express two receptors for BAFF and APRIL, B-cell maturation antigen (BCMA) and TACI [139]. This suggests a role for these molecules in the pathology of MM the disease [127].

In summary, constitutive activation of NF-κB appears to be a frequent event in many haematological malignancies suggesting a pivotal role in the initiation and maintenance of these cancers. It is also apparent that this family of transcription factor can modulate tumour cell responses to chemotherapeutic drugs. The precise mechanisms of NF-κB-mediated drug resistance are not yet fully elucidated, but studies have identified a consensus NF-κB binding site in the human multidrug resistance gene 1 (MDR1) and found that, at least in vitro, NF-κB transactivates the expression of MDR1 [140]. This may result in the enhanced efflux of MDR1 substrates, including doxorubicin, etoposide, paclitaxel, tamoxifen, vincristine and topotecan [141]. In addition, NF-κB is known to regulate the transcription of a number of anti-apoptotic genes including BCL2, BCLXL, BCL2 family member 1/anti-apoptosis protein 1 (BFL1/A1) as well as inhibitor of apoptosis proteins (IAPs) and the caspase-8 regulating protein c-FLIP [142,143,144,145], again supporting the development and/or maintenance of those cancer hallmarks associated with tumour survival. Therefore, targeting NIK-IKKα-NF-κB signalling may reverse drug resistance to standard chemotherapy. Although there are many examples of promising anti-NF-κB agents in pre-clinical development, the challenge is to translate this promise into clinical efficacy. Given that NF-κB plays a critical role in the activation of innate and adaptive immune responses, long-term use of pan-NF-κB inhibitors may not be desirable and may shift attention to emerging IKKα and NIK targeted kinase inhibitors and may prove to be very useful additions to the anti-cancer therapeutic armoury.

## 5. Targeting the IKKα and NIK Protein Kinases with Small Molecule Inhibitors

IKKα and NIK are members of the protein kinome that catalyse the transfer of the terminal phosphate of ATP to substrates containing a serine or threonine residue. Like all kinases, they are arranged into two subdomains that fold into a bi-lobed catalytic core structure with ATP binding in a deep cleft located between the lobes [146,147,148]. The adenine ring of ATP forms two hydrogen bonds with the kinase “hinge” (the segment that connects the *N*- and *C*-terminal kinase domains), with the ribose and triphosphate groups binding in a hydrophilic channel extending to the substrate binding site that features conserved residues that are essential to catalysis. An important amino acid in the ATP-binding site is the gatekeeper (gk) residue. The size of the gatekeeper’s side chain limits the access to the hydrophobic region behind it and defines the potential inhibitor selectivity of the ATP site. The amino acids forming hydrogen bonds with the adenine of ATP (and putative inhibitors) in the hinge region are referred to as gk+1 and gk+3 relative to the position of the gatekeeper [146,149,150]. 

Most kinase inhibitors discovered to date are ATP competitive, typically by forming 1–3 hydrogen bonds with the hinge region residues and through hydrophobic interactions in and around the region occupied by ATP. This class of compounds are referred to as Type I kinase inhibitors and usually occupy the adenine region, the hydrophobic region behind the gk residue, the ribose region and the phosphate-binding region. Type 1 inhibitors are very abundant, which may be a consequence of many compounds being synthesised to mimic the ATP pharmacophore and assessed using enzymatic assays using kinases in their activated conformations [149,151]. 

Type II kinase inhibitors occupy a hydrophobic allosteric site adjacent to the ATP pocket created when the kinase is in its inactive conformation, although they can also extend into the adenine region and form one or two hydrogen bonds with kinase hinge residues in a manner similar to that of type I inhibitors. The amino acids surrounding the allosteric pocket are less conserved relative to those in the ATP binding pocket, which may account for the greater selectivity often achieved with these type II inhibitors [149]. 

There are another two additional types of kinase inhibitors: allosteric and covalent compounds. Allosteric inhibitors bind outside the ATP-binding site, modulating kinase activity from a remote binding point [152], whilst covalent inhibitors form an irreversible bond to the kinase active site, most frequently by reacting with a nucleophilic cysteine residue [153]. 

### 5.1. Development of Small Molecule Inhibitors of IKKα and NIK

Over the past twenty years, many pharmaceutical companies and academic groups have initiated drug discovery projects to target the IKKs, mainly against the IKKβ subunit in the canonical NF-κB pathway [7,154]. While numerous compounds that potently and selectively inhibit this kinase have been published, problems of toxicity have been reported. For example, there is evidence that long-term inhibition of IKKβ can induce significant cell death and apoptosis in normal epithelial cells and cardiac cells respectively [155,156]. The influential role of canonical NF-κB signalling in immune system regulation means there are also concerns associated with the prolonged use of IKKβ inhibitors [157]. Furthermore, Rel A deficient mice exhibit embryonic lethality after around 15 days of gestation and it is thought that this is due to liver degeneration by programmed cell death [158]. Whilst this is only observed in knockout mice, a small population of the Cree people in Canada have been found to carry a mutation which leaves them IKKβ null and severely immunocompromised, leading to death usually within months of birth [159]. These observations have raised the concern that excessive IKKβ inhibition will result in hepatic toxicity and immunosuppression [160]. 

**Figure 4:**
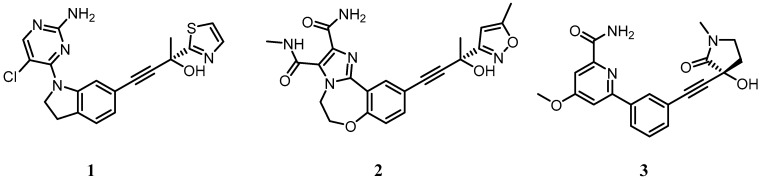
Small molecule inhibitors of NIK developed by Amgen and Genentech. For full chemical names of **1**–**3** see Appendix A.

The absence of reports of toxicity from non-canonical NF-κB inhibitors is primarily due to the fact that so few of them have been published and expansive toxicological data has not yet been acquired. Developing inhibitors against the non-canonical NF-κB pathway has its challenges, particularly as IKKα plays a role in the canonical NF-κB pathway [161,162] and is also known to be involved in the phosphorylation of IKKβ in some settings [121]. Its roles independent of the NF-κB signalling pathway described earlier will also be impaired if it is inhibited as a phosphorylating entity. This may have advantages in a cancer setting as multiple roles of IKKα may be involved, but if discrimination between non-canonical NF-κB signalling and other IKKα outputs is the aim, inhibiting its kinase function may not be the answer, and targeting NIK may be the preferred route. NIK phosphorylates IKKβ to a much lesser extent than IKKα and has far fewer roles outside the non-canonical NF-κB pathway [121]. Furthermore, from a drug discovery perspective, a NIK inhibitor with no canonical NF-κB activity may be more tractable than a specific IKKα inhibitor due to the high level of homology between IKKα and IKKβ. Despite this, there have been very few disclosures of NIK inhibitors in the literature. This may be because X-ray crystallographic studies with the NIK kinase domain have revealed a constrained ATP binding site with a narrow channel to the back pocket behind the gk, rendering the in-building of selectivity over other kinases challenging [163,164]. Amgen and Genentech have pioneered small molecule inhibitor development against NIK using a propargyl alcohol as a common motif to access the hydrophobic site behind the gk (e.g., compounds **1** and **2**; Figure 4), and a synopsis of their approach is covered comprehensively in a recent publication by Castanedo [165] (and references therein). While large improvements in potency and kinase selectivity were obtained with the development of compound **2**, it was insufficiently robust for in vivo evaluation of NIK pharmacology. A recent publication by the same group [166] has reported compound **3** (Figure 4), a highly potent and selective NIK inhibitor with suitable properties for advanced ADME and pharmacology experiments that will prove to be a useful tool to dissect the roles of NIK and IKKα with respect to the non-canonical NF-κB pathway in cancer.

Here we review the development of selective small molecule inhibitors of IKKα, which is an attractive target in prostate and other cancers, as we describe in this chapter and is the primary focus of research in our laboratories.

### 5.2. Structure of IKKα and IKKβ from a Small Molecule Design Perspective

The predominant form of the IKK complex (700 to 900 kDa) that is associated with canonical NF-κB signalling includes two catalytic subunits, IKKα and IKKβ, the regulatory subunit NEMO (or IKKγ) and IKAP [167], Rap1 [168], heat shock protein 90 (Hsp90) or Cdc37 [161]. IKKα has also been identified as a homodimeric complex, and more recently, as a trimer of dimers, that may be involved in non-canonical NF-κB signal induction [169]. IKKα and IKKβ share 52% amino acid identity with a similar predicted structural organisation, which includes an *N*-terminal kinase domain (KD), kinase-binding domain (ΚBD), three coiled coil regions (CC1-3), a zinc finger domain, a centrally positioned leucine-zipper motif (LZ) (involved in the dimerisation process) and a C-terminal helix-loop-helix domain (HLH) followed by a NEMO-binding domain (NBD) (involved in NEMO interaction when unphosphorylated) ([170]; see IKKα schematic Figure 2A). The KD of both IKK isoforms contains an activation loop with closely spaced serine residues at positions 176 and 180 in IKKα and positions 177 and 181 in IKKβ [167]. IKKβ presents an additional ubiquitin-like domain (ULD) following the KD, and conserved buried residues in this region and of the ULD–SDD (scaffold dimerisation domain) interface suggests that IKKα also has this domain [171]. IKKβ crystal structures have shown that LZ and HLH motif are part of an elongated α-helical SDD located after the ULD. Both the KD and the ULD interact with the SDD forming a trimodular unit [171,172,173]. 

**Figure 5:**
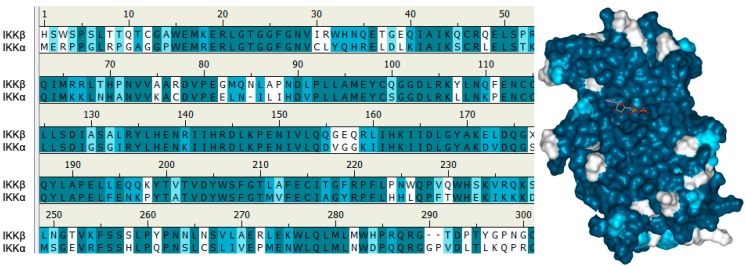
**Left**: Sequence alignment for the kinase domains of IKKα and IKKβ showing 61% of identical residues (coloured in turquoise), a further 14% similar residues (polar for polar, hydrophobic for hydrophobic; in light blue) and 25% non-similar residues (white). X at positions 177 and 181 in IKKβ represent the residues in the activation loop of IKKβ that have been crystallised containing Ser or phosphoserine residues in the inactive or active conformations respectively. **Right**: Structure of IKKβ highlighting residues that are identical to (turquoise), similar to (light blue) or different from (white) IKKα. The ATP analogue marks the ATP binding site and is surrounded by turquoise residues.

When superimposing the primary sequences of the KDs of IKKα and IKKβ, it is striking to see the level of homology between the two isoforms (Figure 5). Furthermore, in the ATP binding site, residues are almost completely identical (both have Met as the gk residue, with gk+1 and gk+3 as Glu and Cys respectively), with the exception of Gln100 in the hinge binding region of IKKβ being replaced by Ser in IKKα. A three-dimensional representation of the kinase domain of IKKβ (using the 4KIK.pdb chain B crystal structure [173]) that includes a molecule of ATP highlights this sequence alignment with IKKα and clearly shows how similar the ATP-binding sites are likely to be in these two isoforms [28]. Nevertheless, given that a number of structurally diverse compounds that are selective for IKKβ over IKKα have been reported [7,153], the implication is that selective inhibitors for the latter isoform should be possible. Whilst crystal structures for IKKβ have been reported in both its inactive and active form (with phosphorylated Ser residues in the activation loop) [171,172,173], these structures have not yet been able to explain how either IKKα or IKKβ, in response to different upstream stimuli, act to regulate such a wide range of canonical and non-canonical NF-κB-dependent activities. To date, no group has been able to successfully crystallise IKKα and report a high-resolution structure of sufficient detail to guide structure-based inhibitor design. Using a combination of X-ray crystallography and single-particle cryoelectron microscopy (cryo-EM), Polley and coworkers [169] have generated structures of IKKα in dimeric (~150 kDa) and hexameric (~450 kDa) forms and identified a unique surface in the SDD and KD interface that could be responsible for interacting with NIK and p100, which is absent in IKKβ. They argue that this unique surface could be targeted to prevent its assembly with these proteins in order to selectively block the activation of NF-κB through the non-canonical pathway, although there are no reports of agents targeting this region to date. Furthermore, there was insufficient structural detail associated with this 3-dimensional IKKα structure to identify differences in the ATP-binding site of IKKβ to direct the design of small molecule competitive inhibitors to selectively target this region.

A number of studies have shown that both IKKs have high affinity (Km, 100–600 nM) for ATP [174,175], which can pose significant challenges for ATP binding site inhibitors in view of the high levels of cellular ATP with which they have to compete [176,177]. The binding mode of the staurosporine analogue K252a in the crystal structure complex with IKKβ [172] is virtually identical regardless of the phosphorylation state of the KD, suggesting that unless inhibitors can be developed that are able to differentiate between the active and inactive kinase conformations (e.g., allosteric non-competitive ATP inhibitors), recapitulating potent activity in a cellular environment may be difficult to achieve. Nevertheless, the large number of potent IKKβ inhibitors that have been reported in the literature to date, which bind in or close to the ATP-binding site and recapitulate activity against cell-based pharmacodynamic readouts for IKKβ at sub-micromolar concentrations [7,154] suggests that IKKα is also a druggable target.

### 5.3. IKKα/β Non-Selective Inhibitors

There are more than 150 patents and papers specifically associated with small molecule IKK inhibitor identification and development (IKKα, IKKβ or the whole IKK complex) reported in the scientific literature, including synthetic and natural products ranging from simple molecules like aspirin [178] to complex structures like staurosporine [179]. The majority of these compounds show selectivity for IKKβ over IKKα and block numerous responses based on the role of the canonical IKKβ/NF-κB pathway. Many marine and terrestrial natural products have been identified as modulators of NF-κB [180], although only a few like artemisolide [181], honokiol [182], wedelolactone [183] or geldanamycin [184] can directly target the IKK complex or its subunits. 

**Figure 6:**
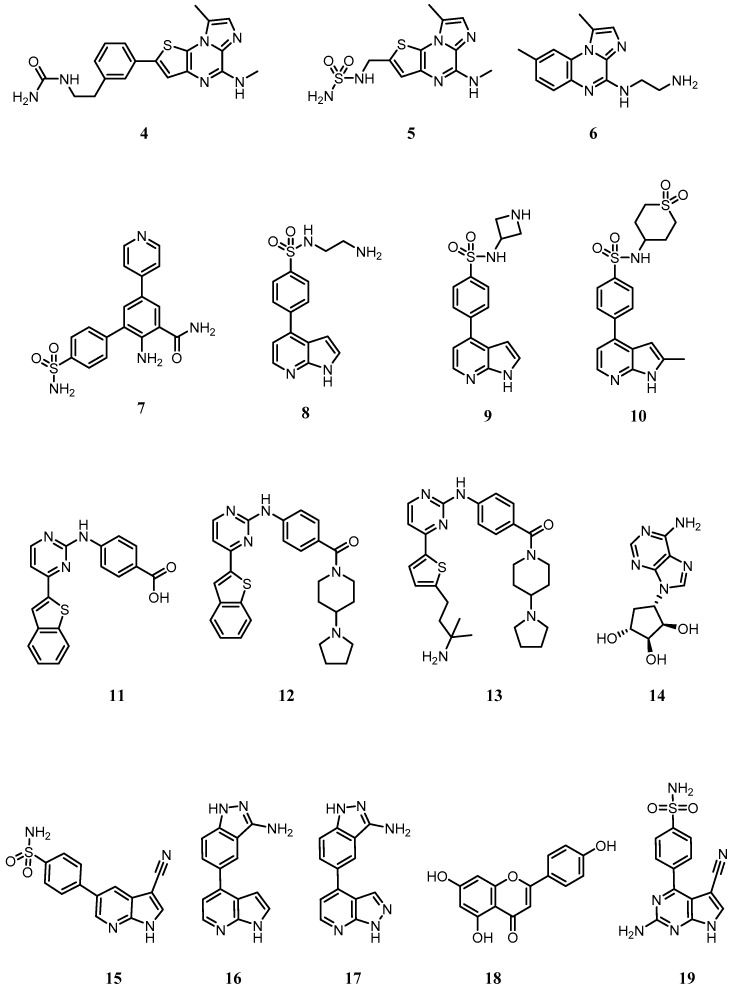
Small molecule dual inhibitors of IKKα and IKKβ or selective for IKKα alone. For full chemical names of **4**–**19** see Appendix A.

Despite the scarcity of information concerning IKKα inhibitors in the drug discovery literature, a few groups have reported data on potent but non-selective IKKα inhibitors. The following description is focused on identifying structural trends around IKKα inhibition (Figure 6), notwithstanding that the compounds themselves were originally designed to target IKKβ. 

In a series of imidazo(1,2-*a*)thieno(3,2-*e*)pyrazines developed by Belema as IKKβ inhibitors [185], urea or sulfonyl urea substituents at the 2-position of the thiophene ring were found to promote IKKα activity (Compounds **4** and **5**, IC_50_s ~ 0.15 µM; Figure 6). No assumptions on the binding mode of these molecules can be made due to a potential allosteric mode of action suggested by previous work on related compound **6** (BMS-345541; Figure 6) [186]. Indeed, BMS-345541 has often been used to explore the role of IKKα in cell signalling and phenotypic studies, but its inhibition of IKKβ at similar concentrations means that any cell-based effects observed cannot be attributed to IKKα inhibition alone.

In 2007, Christopher reported a series of 2-amino-3,5-diarylbenzamides as non-selective IKKα/β inhibitors (Figure 6) [187,188]. The primary amine/carboxamide functionalities in this class of compounds were essential to achieve IKKα and IKKβ µM activity. A 4-sulfamoylbenzene ring at the 3-position and a 4-pyridyl substituent at the 5-position revealed compound **7** as the most active compound against IKKα (IC_50_ = 0.079 µM). No mention of the role of the 4-pyridyl substituent is made, but replacement with a phenyl ring induced a notable drop in IKKα activity (IC_50_ = 1.0 µM), suggesting a key interaction of the heterocyclic nitrogen with the ATP site in IKKα. Interestingly, it was a compound from this series that was used in the IKKα structure reported using single-particle cryo-EM by Polley [169]. 

In 2009, another GlaxoSmithKline team identified 4-phenyl-7-azaindoles as IKKβ selective inhibitors (Figure 6) [189]. Within this series, compound **8** also showed good activity against IKKα (IC_50_ = 0.251 µM), with the azetidine **9** showing improved potency (IC_50_ = 0.040 µM). Replacement of the azetidine ring with a cyclic sulfone improved IKKα activity even further (Compound **10** IC_50_ = 0.013 µM), although potent activity against IKKβ was retained. Docking studies suggested the pyrrole and the pyridine nitrogens are involved in hydrogen bonding with the hinge region of both isoforms and the sulfonamide oxygens are thought to be hydrogen bonding with Lys44 at the back of the adenine cavity in IKKβ.

Waelchli [190] reported 2-benzamidopyrimidines as IKKβ inhibitors (Figure 6) and identified that compound **11** with a carboxylic acid showed moderate IKKα activity (IC_50_ = 5 µM). Conversion to amide functionalities revealed that compound **12** improved IKKα inhibition (IC_50_ = 0.1 µM), whilst replacement of the benzothiophene group with a 5-substituted thiophene led to an even better, but equally non-selective, IKKα inhibitor (Compound **13**; IC_50_ = 0.03 µM). 

During 2008 the first IKKα selective inhibitors were identified. Asamitsu [191] reported that the natural product noraristeromycin (NAM; **14** (Figure 6), a known carbocyclic adenine nucleoside with anti-trypanosomal activity was an NF-κB inhibitor (IC_50_ 2.7 µM) which blocked TNF-α-induced IκBα phosphorylation and degradation in cells, prevented p65 phosphorylation and showed a strong suppressive effect on HIV Type 1 replication in chronically infected OM10.1 cells upon TNF-α stimulation. NAM was selected from a panel of related carbocyclic adenine nucleoside derivatives such as 2-fluoro, 2-bromo and 2-amino-NAM, which showed similar inhibition of NF-κB-mediated transcription but a greater cytotoxic effect. However, as the pharmacodynamic readouts used are reporters for both IKKβ and IKKα activity in cells (IκBα phosphorylation and p65 phosphorylation) and do not focus on specific biomarkers of the IKKα-controlled non-canonical NF-κB pathway, such as p100 phosphorylation and subsequent processing to p52, it is difficult to characterise this agent as truly IKKα-selective.

Christopher from GlaxoSmithKline filed a short international patent on March 2008 which claimed a series of 33 pyrrolopyridines as IKKα inhibitors [192]. The heterocyclic core was substituted at the 5-position with a nitrile and various aryl groups at the 3-position, most of which contained polar substituents such as sulfonamides or various hydrogen bond donor motifs as seen in compound **15** (Figure 6). Many of these compounds were reported as sub-µM IKKα inhibitors and some were claimed to be active against IKKβ, JNK-1 and P13Kγ. Their use outside of this patent has not been reported.

In a concise subsequent patent, Christopher presented compounds **16** and **17** (Figure 6), but without any specific biological data other than stating that both compounds were said to inhibit IKKα and IKKβ with IC_50_ < 1 µM, as well as showing inhibitory activity against ROCK1 and YAK3 kinases [193]. Again, their use outside of this patent has not been reported.

More recently, Gupta [194,195] have explored the role of the natural product apigenin (compound **18**; Figure 6) as an inhibitor of IKKα and the NF-κB pathway in prostate cancer. Although they demonstrated that apigenin had a higher binding affinity for IKKα than IKKβ in PC3 cells (although affinity was not quantified), they concluded that the reduction in prostate cancer growth in vitro and in vivo was a consequence of the suppression of NF-κB/p65 activation via inhibition of both isoforms, suggesting that apigenin is not a selective IKKα inhibitor in cells. 

To date, the only selective IKKα inhibitors reported that recaptitulate activity in cells against recognised pharmacodynamic markers associated with the non-canonical NF-κB pathway have emerged from our own laboratories [28]. Using a homology model of the IKKα kinase domain based on the crystal structure of IKKβ (chain B, residues 1–309, PDB entry 4KIK [173]) and performing MD simulations and analysing the descriptors of motion extracted from their MD trajectories such as residual fluctuation, we revealed dynamic differences between the two isoforms that could be exploited in an inhibitor design programme. The recent publication of the low-resolution IKKα structure by cryoEM [169] confirmed our structural analysis through our homology model [28]. Overall, the two isoforms behaved very similarly, but IKKα appeared more flexible in several key areas around the ATP binding site, particularly at the G-loop above the site entrance and the loop located just adjacent to the hinge (residues 155–159 in IKKα (VGGKI) and residues 156–160 in IKKβ (GEQRL) (Figure 5). 

Based on these key dynamic differences in the ATP-binding site, we designed and synthesised the first example of selective inhibitors of IKKα. Crucially, our compounds also demonstrated effective target engagement and selectivity with IKKα; in U2OS cells SU909 (compound **19**) inhibited IKKα-driven p100 phosphorylation in the non-canonical NF-κB pathway without affecting IKKβ-dependent IκBα loss in the canonical NF-κB pathway [28]. Whilst SU909 was not particularly potent in the cell-based environment (IC_50_ = 8 μM against agonist-stimulated p100 phosphorylation), it has enabled further compound optimisation to produce highly potent and selective inhibitors with submicromolar activities in cells (manuscript in preparation). 

## 6. Future Perspectives

Selective inhibitors of IKKβ catalytic activity have been by far the main focus of the pharmaceutical industry in an attempt to modulate aberrant NF-κB signalling and GlaxoSmithKline has been the key player in the IKK arena. However, only a handful of compounds have been advanced into the early clinical stages and the lack of clinical success has diminished the interest of the pharmaceutical industry in inhibitors of this isoform. Nevertheless, the availability of these inhibitors with a variety of chemical scaffolds that have different kinome selectivity profiles means that researchers in the IKKβ/NF-κB canonical pathway field have many pharmacological tools with which to explore it. IKKα is the isoform that has received less attention from the research community in terms of inhibitor development though as described this is evolving. With parallel development of NIK inhibitors, it offers the opportunity to target NIK-IKKα signalling in a subtly different manner. IKKα can generate a gene expression pattern distinct from IKKβ which offers an interesting alternative given the lack of success of the IKKβ inhibition approach to date. Kinase inhibitors with isoform selectivity will allow further refinement of the different roles of NIK, IKKα and β in mediating transcriptional responses in a variety of settings. The consequences of pharmacological inhibition of the kinase activity of NIK and IKKα, particularly in an in vivo setting, should identify if these targets have advantages in different tumour settings, and offer a route to the clinic.

Kinase inhibitors of this nature will enable many diverse research questions to be addressed. Basic experiments such as defining substrate specificities through to more complex investigations of functional roles in vivo will likely follow but a few key questions do stand out. For example, will these kinase inhibitors enable reappraisal of the functional role(s) of NIK, IKKα and β previously identified by means of molecular perturbation/modulation of NIK and IKKs? Both over-expression (of wild-type and mutant forms) and deletion/run-down strategies have undoubtedly identified potential functional roles for these kinases however these techniques fundamentally change the expression levels of the kinases in any cellular setting. It remains to be determined to what extent the make-up of multi-isoform IKK complexes is changed following molecular interventions compared to direct inhibition by small molecule inhibitors. Comparison of the effects of kinase inhibitors versus molecular ‘run-down’ strategies will further clarify what are kinase-dependent events relative to those that are perhaps kinase-independent such as scaffolding roles for each kinase. Furthermore, by examining the impact upon individual substrates and their associated downstream signalling these inhibitors may also help delineate IKKα-dependent NF-κB-dependent cellular responses versus those that are IKKα-mediated but independent of NF-κB signalling. Another question outstanding is to what extent does IKKα function in the canonical NF-κB pathway and does pharmacological targeting of IKKα bring undesirable modulation of p65/p50 activity and pathway cross-talk resulting in the toxicities displayed with IKKβ inhibitors? 

It seems likely that medicinal chemistry efforts in academia will drive research against IKKα and NIK over the coming years, providing innovative pharmacological approaches to what could be viewed as high-risk projects. That said, availability of effective inhibitors of IKKα and NIK will over time enable a better understanding of the cellular signalling events and gene induction profiles driven by IKKα and NIK. For example, in diverse solid tumour and haematological settings described above, as well as more diverse tumour types not considered here, these molecules will give insight into whether intervention against these targets can generate positive outcomes whilst avoiding potential as yet lesser known toxicities. 

## Appendix A

List of chemical names for compounds **1**–**19**	
**1**	(*R*)-4-(1-(2-amino-5-chloropyrimidin-4-yl)indolin-6-yl)-2-(thiazol-2-yl)but-3-yn-2-ol
**2**	(*R*)-10-(3-hydroxy-3-(5-methylisoxazol-3-yl)but-1-yn-1-yl)-N3-methyl-5,6-dihydrobenzo[f]imidazo[1,2-*d*][1,4]oxazepine-2,3-dicarboxamide
**3**	(*R*)-6-(3-((3-hydroxy-1-methyl-2-oxopyrrolidin-3-yl)ethynyl)phenyl)-4-methoxypicolinamide
**4**	1-(3-(8-methyl-5-(methylamino)imidazo[1,2-*a*]thieno[3,2-*e*]pyrazin-2-yl)phenethyl)urea
**5**	*N*-((8-methyl-5-(methylamino)imidazo[1,2-*a*]thieno[3,2-*e*]pyrazin-2-yl)methyl)aminosulfonamide
**6**	*N*-(1,8-dimethylimidazo[1,2-*a*]quinoxalin-4-yl)ethane-1,2-diamine
**7**	2-amino-5-(pyridin-4-yl)-4’-sulfamoyl-[1,1’-biphenyl]-3-carboxamide
**8**	*N*-(2-aminoethyl)-4-(1*H*-pyrrolo[2,3-*b*]pyridin-4-yl)benzenesulfonamide
**9**	*N*-(azetidin-3-yl)-4-(1H-pyrrolo[2,3-*b*]pyridin-4-yl)benzenesulfonamide
**10**	*N*-(1,1-dioxidotetrahydro-2*H*-thiopyran-4-yl)-4-(2-methyl-1*H*-pyrrolo[2,3-*b*]pyridin-4-yl)benzenesulfonamide
**11**	4-((4-(benzo[*b*]thiophen-2-yl)pyrimidin-2-yl)amino)benzoic acid
**12**	4-((4-(benzo[*b*]thiophen-2-yl)pyrimidin-2-yl)amino)phenyl)(4-(pyrrolidin-1-yl)piperidin-1-yl)methanone
**13**	4-((4-(5-(3-amino-3-methylbutyl)thiophen-2-yl)pyrimidin-2-yl)amino)phenyl)(4-(pyrrolidin-1-yl)piperidin-1-yl)methanone
**14**	(1*R*,2*S*,3*R*,4*S*)-4-(6-amino-9*H*-purin-9-yl)cyclopentane-1,2,3-triol (NAM)
**15**	4-(3-cyano-1*H*-pyrrolo[2,3-*b*]pyridin-5-yl)benzenesulfonamide
**16**	5-(1*H*-pyrrolo[2,3-*b*]pyridin-4-yl)-1*H*-indazol-3-amine
**17**	5-(1*H*-pyrazolo[3,4-*b*]pyridin-4-yl)-1*H*-indazol-3-amine
**18**	5,7-dihydroxy-2-(4-hydroxyphenyl)-4*H*-chromen-4-one (apigenin)
**19**	4-(2-amino-5-cyano-7*H*-pyrrolo[2,3-*d*]pyrimidin-4-yl)benzenesulfonamide (SU909)
